# Quantitative Proteomics Reveals Myosin and Actin as Promising Saliva Biomarkers for Distinguishing Pre-Malignant and Malignant Oral Lesions

**DOI:** 10.1371/journal.pone.0011148

**Published:** 2010-06-17

**Authors:** Ebbing P. de Jong, Hongwei Xie, Getiria Onsongo, Matthew D. Stone, Xiao-Bing Chen, Joel A. Kooren, Eric W. Refsland, Robert J. Griffin, Frank G. Ondrey, Baolin Wu, Chap T. Le, Nelson L. Rhodus, John V. Carlis, Timothy J. Griffin

**Affiliations:** 1 Department of Biochemistry, Molecular Biology and Biophysics, University of Minnesota, Minneapolis, Minnesota, United States of America; 2 Department of Computer Science and Engineering, University of Minnesota, Minneapolis, Minnesota, United States of America; 3 Department of Otalaryngology, University of Minnesota, Minneapolis, Minnesota, United States of America; 4 Department of Biostatistics, University of Minnesota, Minneapolis, Minnesota, United States of America; 5 Department of Oral Medicine, Diagnosis and Radiology, University of Minnesota, Minneapolis, Minnesota, United States of America; 6 Guanghua School of Stomatology, Sun Yat-Sen University, Guangzhou, China; 7 Department of Radiation Oncology, University of Arkansas for Medical Sciences, Little Rock, Arkansas, United States of America; National Cancer Institute, United States of America

## Abstract

**Background:**

Oral cancer survival rates increase significantly when it is detected and treated early. Unfortunately, clinicians now lack tests which easily and reliably distinguish pre-malignant oral lesions from those already transitioned to malignancy. A test for proteins, ones found in non-invasively-collected whole saliva and whose abundances distinguish these lesion types, would meet this critical need.

**Methodology/Principal Findings:**

To discover such proteins, in a first-of-its-kind study we used advanced mass spectrometry-based quantitative proteomics analysis of the pooled soluble fraction of whole saliva from four subjects with pre-malignant lesions and four with malignant lesions. We prioritized candidate biomarkers via bioinformatics and validated selected proteins by western blotting. Bioinformatic analysis of differentially abundant proteins and initial western blotting revealed increased abundance of myosin and actin in patients with malignant lesions. We validated those results by additional western blotting of individual whole saliva samples from twelve other subjects with pre-malignant oral lesions and twelve with malignant oral lesions. Sensitivity/specificity values for distinguishing between different lesion types were 100%/75% (p = 0.002) for actin, and 67%/83% (p<0.00001) for myosin in soluble saliva. Exfoliated epithelial cells from subjects' saliva also showed increased myosin and actin abundance in those with malignant lesions, linking our observations in soluble saliva to abundance differences between pre-malignant and malignant cells.

**Conclusions/Significance:**

Salivary actin and myosin abundances distinguish oral lesion types with sensitivity and specificity rivaling other non-invasive oral cancer tests. Our findings provide a promising starting point for the development of non-invasive and inexpensive salivary tests to reliably detect oral cancer early.

## Introduction

Oral cancer develops in stages, transitioning from a normal oral epithelium, to a pre-malignant, dysplastic oral lesion, to a malignant lesion, most commonly in the form of oral squamous cell carcinoma (OSCC). For those who develop OSCC, the overall 5- year survival rate is approximately 50%, unchanged over the last 30 years[1]. For those where malignancy is detected early, soon after transitioning from pre-malignancy, treatment is more effective, and consequently the survival rate increases to about 80%[2]. Clearly, the ability to distinguish between pre-malignant and malignant oral lesions is crucial[1], [3].

Unfortunately, pre-malignant and malignant lesion types cannot be distinguished simply by visual inspection; instead invasive tests are used. The current gold standard for characterizing lesions, histological analysis of tissue biopsies[3], has several disadvantages: expert clinicians are required to collect the samples and interpret results, incurring relatively high costs; inaccuracies in diagnosis due to difficulties sampling tissue which may have multiple dysplastic foci; and patient discomfort with the procedure. Together these disadvantages limit the accuracy of diagnosis, the frequency of patient testing, and consequently the ability to detect oral cancer early by tissue biopsy.

Non-invasive, inexpensive and accurate tests, ones which foster early and frequent screening, would, if available, transform the early detection of oral cancer. Whole saliva collection is non-invasive, providing inexpensive collection of ample amounts of sample for analysis in an on-demand manner[4], [5]. Protein biomarkers in whole saliva could fulfill this need if their abundance levels distinguish patients with pre-malignant oral lesions from those with malignant lesions. Whole saliva's direct interaction with the oral lesion points to a high probability of it containing lesion-associated proteins diagnostic of its pre-malignant or malignant status. Ideally, protein biomarkers from saliva would be detected inexpensively and easily using point-of-care devices, either in the clinic or even at home.

So far there are only a few proteomic studies relevant to developing such protein-based tests. Analyzing biopsied tissues, two such studies were recently published by Siu and colleagues, resulting in the identification of several promising biomarkers. One study compared protein abundance levels between healthy tissue and pre-malignant dysplastic tissue[6]; the other compared protein abundance levels between healthy tissue and malignant tissue[7]. Another study identified proteins whose abundance differed between healthy and malignant oral tissue, and then validated, via immunohistochemistry, the ability of some of these proteins to distinguish pre-malignant dysplastic lesions from malignant lesions[8]. However, the potential biomarker proteins were not validated in saliva, where they would have the most value for clinical assays. Another recent study in saliva[9] used proteomics to discover proteins whose abundance levels distinguished subjects with no oral lesions from those with malignant lesions.

Sadly, given their great potential for discovering promising biomarkers for early detection of oral cancer, no proteomic studies in saliva have been undertaken comparing subjects with pre-malignant and malignant lesions. Despite the urgent need for such studies, these are not easily undertaken. A main reason for this is the difficulty in collecting even modest numbers of samples from subjects with pre-malignant oral lesions, as most clinicians see these subjects sporadically and lack coordinated infrastructure for sample collection and analysis.

In this first-of-its-kind study, we have overcome this difficulty, collecting saliva samples from subjects with pre-malignant oral lesions and using advanced mass spectrometry-based proteomics to discover salivary protein abundance differences between these subjects and those with malignant oral lesions. Via bioinformatic analysis and biochemical validations, we have identified myosin and actin as promising salivary biomarkers capable of distinguishing between subjects with pre-malignant and malignant lesions. Our results provide a basis for the potential development of a salivary-based test for the early detection of oral cancer.

## Materials and Methods

### Ethics statement

The study was done with informed written consent of all sample donors using a human subjects protocol approved by the Institutional Review Board at the University of Minnesota (IRB study number 0001M34501).

### Clinical whole saliva collection protocol from subjects

Unstimulated whole saliva was collected from two groups: subjects with pre-malignant dysplastic lesions diagnosed by scalpel biopsy and histology, and subjects with invasive malignant lesions, diagnosed as primary OSCC by scalpel biopsy and histology. All OSCC patients were non-metastatic, and had either T1 or T2 stage tumors. Subjects with either lesion type (pre-malignant or malignant) had not undergone any treatment or surgery (including tissue biopsy) at the lesion site prior to saliva collection. Unstimulated whole saliva was obtained using a standard, controlled protocol[10] by first having each subject swallow and then expectorate continuously into a 50-ml sterile, polypropylene conical tube for a period of 5 min. This resulted in about 2–5 mL of total saliva from each subject. Following collection, the samples were immediately placed on ice and stored at −70°C until further processing. Subjects included in the study were free of confounding conditions: periodontal disease, auto-immune disease, a prior history of diseases of the oral mucosa, or current use of potentially confounding medications.

### Whole saliva sample preparation

For proteomic discovery studies and western blot validation studies, samples from subjects falling in the two groups were prepared identically. Whole saliva was centrifuged at 3000 x g at 4°C, the supernatant containing the soluble fraction of saliva proteins removed, and the cell pellets washed with PBS and lysed to obtain cellular proteins, as previously described[11]. Total protein was quantified using the BCA assay (Thermo Pierce).

### Protein Digestion, iTRAQ labeling and peptide fractionation

For proteomic discovery experiments, 200 µg of denatured and reduced protein from the soluble portion of saliva from each of four subjects with pre-malignant lesions was pooled together; likewise, 200 µg of denatured and reduced protein from the soluble portion of saliva from each of four subjects with pre-malignant lesions was pooled together. Each separate sample pool was digested overnight with trypsin as previously described[11], [12]. Resulting peptides were concentrated and purified via mixed-mode cation exchange solid-phase extraction columns (Waters). The resulting peptides from each of the subjects were then combined and labeled with the iTRAQ reagent[13] (Applied Biosystems, Foster City) following the manufacturer's procedure, labeling samples from subjects with pre-malignant lesions with reagent 115 and samples from subjects with malignant lesions with reagent 116. The iTRAQ labeled peptide mixtures were combined and fractionated using a 3-step method described previously combining preparative isoelectric focusing (IEF), strong cation exchange (SCX) and reversed-phase microcapillary liquid chromatography[11].

### Mass spectrometric analysis

Mass spectrometry was done using an LTQ linear ion trap mass spectrometer (Thermo Finnigan, Inc) using Xcalibur version 2.0 operating software. In order to detect low mass reporter ions from the iTRAQ reagent, the instrument was operated using pulsed Q dissociation (PQD) mode. We have described in detail the procedure used for PQD analysis of iTRAQ reagent labeled peptides previously, as well as the HPLC and LTQ operating conditions for tandem mass spectrometry (MS/MS) analysis[14].

### Protein Identification and Quantification

MS/MS spectra were searched using SEQUEST(15) (Version 27, Thermo Finnigan, San Jose, CA) against a non-redundant human protein sequence database from the European Bioinformatics Institute (ipi.HUMAN.v3.18.fasta, containing 62,000 entries) comprised of both the forward and reversed sequences, using parameters as previously described[11]. Peptides matched to MS/MS by SEQUEST were filtered using their expected isoelectric points, based upon their fractionation by preparative IEF, as we have described previously[11]. The estimated spectral false positive rate for both the soluble and cellular proteins were below 1%, based on matches to the reversed database[15]. In order to report the minimum set of protein sequences in our proteomic catalogue that adequately accounts for all matched peptides we analyzed all matched peptide sequences for redundant inclusion in multiple proteins in our sequence database. Proteins identified from at least one non-redundant peptide sequence were retained in our catalogue. For proteins identified from redundant peptides, we retained those proteins in our catalogue only if the redundant proteins matched to a closely related isoform of the same protein in the database. Proteins identified from redundant peptides that matched to different proteins entirely were removed.

Relative protein abundance ratios between the two subject groups were calculated from iTRAQ reagent reporter ion intensities, using an in-house developed automated software program, as we have previously described[14]. Only proteins identified from two or more MS/MS spectra matched to peptides were considered for quantitative analysis. To account for any systematic errors biasing our relative protein abundance ratios, each protein ratio was normalized by calculating the median ratio across the entire set of proteins and dividing each protein ratio by this median value. Normalization was done by diving each ratio by the median ratio in the log_2_ scale: normalized ratio  = 2^[log^_2_^(protein ratio) - log^_2_^(median ratio)]^.

To determine proteins showing significant abundance differences between the different subject groups, mean and standard deviation for each ratio was calculated across all matched MS/MS spectra, and used to determine proteins showing significant differences in abundance between the two groups. Proteins with abundance differences greater than one standard deviation from the mean value were deemed to be significantly changing.

### Bioinformatic analysis

Proteins from the soluble fraction of whole saliva showing significant abundance differences between the two subject groups were analyzed using Ingenuity Pathway Analysis (IPA) software (Ingenuity Systems, Inc) were mapped to canonical pathways. For each dataset analyzed, a corresponding control dataset was also analyzed containing proteins not showing abundance differences between the two stages of oral cancer development. A relative abundance window centered at unity was used to generate a random dataset of control proteins numbering the same as the proteins showing differences in relative abundance. The significance of the association between proteins within each dataset and the canonical pathway was measured in two ways: 1) A ratio of the number of molecules from the data set that map to the pathway divided by the total number of molecules that map to the canonical pathway is displayed. 2) Fisher's exact test was used to calculate a p-value determining the probability that the association between the genes in the dataset and the canonical pathway is explained by chance alone.

### Western blot validation experiments

Commercial antibodies used for western blot validation experiments were as follows (product numbers in parentheses): myosin regulatory light chain, monoclonal antibody (Santa Cruz Biotechnology, Inc., sc-48414), and actin, monoclonal anti-actin Ab5 (BD Transduction Laboratories, 612657).

Thirty µg of protein from each individual subject analyzed in validation experiments (12 individuals with pre-malignant lesions and 12 with malignant lesions), along with thirty µg of control protein from a control saliva pool, and molecular weight markers was loaded in separate lanes and separated via SDS-PAGE on a Bio-Rad minigel apparatus. The proteins were transferred to a PVDF membrane and the membrane blocked with 1xPBS containing 2.5 mg/ml BSA and 0.25% (v/v) Tween 20, followed by incubation with the primary antibody, diluted in PBS at concentrations suggested by the antibody manufacturer. After washing, the membranes were incubated with secondary antibodies conjugated to horseradish peroxidase (HRP).

For detection, membrane-bound proteins recognized by the HRP conjugated secondary antibody were visualized by chemilumenescence using the SuperSignal West Pico ECL Substrate (Thermo Scientific). Developed films were imaged on a Bio-Rad GS-700 Imaging Densitometer, and quantification performed on background-subtracted bands with Molecular Analyst (v. 21) Software. Densitometric values of all individual samples were normalized against the signal from the common control. All blots from soluble saliva were performed in triplicate, and the mean values were used for statistical analyses.

Image files generated from densitometry detection were imported to Adobe Photoshop version 9.0.2. Images were cropped to show detected bands at correct, expected molecular weight regions, and then converted to grayscale and subjected to automated contrast adjustment using the default program settings.

### Statistical analysis

For each candidate biomarker, the statistical difference between the pre-malignant and malignant samples was compared using the two-sample t-test with the Welch-Satterwaite method for samples of different variances. If the difference is statistically significant at the 5% level, an optimal cut-point is determined at which the Youden's Index is maximized[16]. Sensitivity (1-false negative rate), the probability to correctly identify saliva collected from a subject with a malignant lesion, and specificity (1-false positive rate), the probability to correctly identify saliva collected from a subject with a pre-malignant lesion, were calculated at that optimal cut-point.

## Results

### Discovery of candidate biomarkers

**Figure 1:**
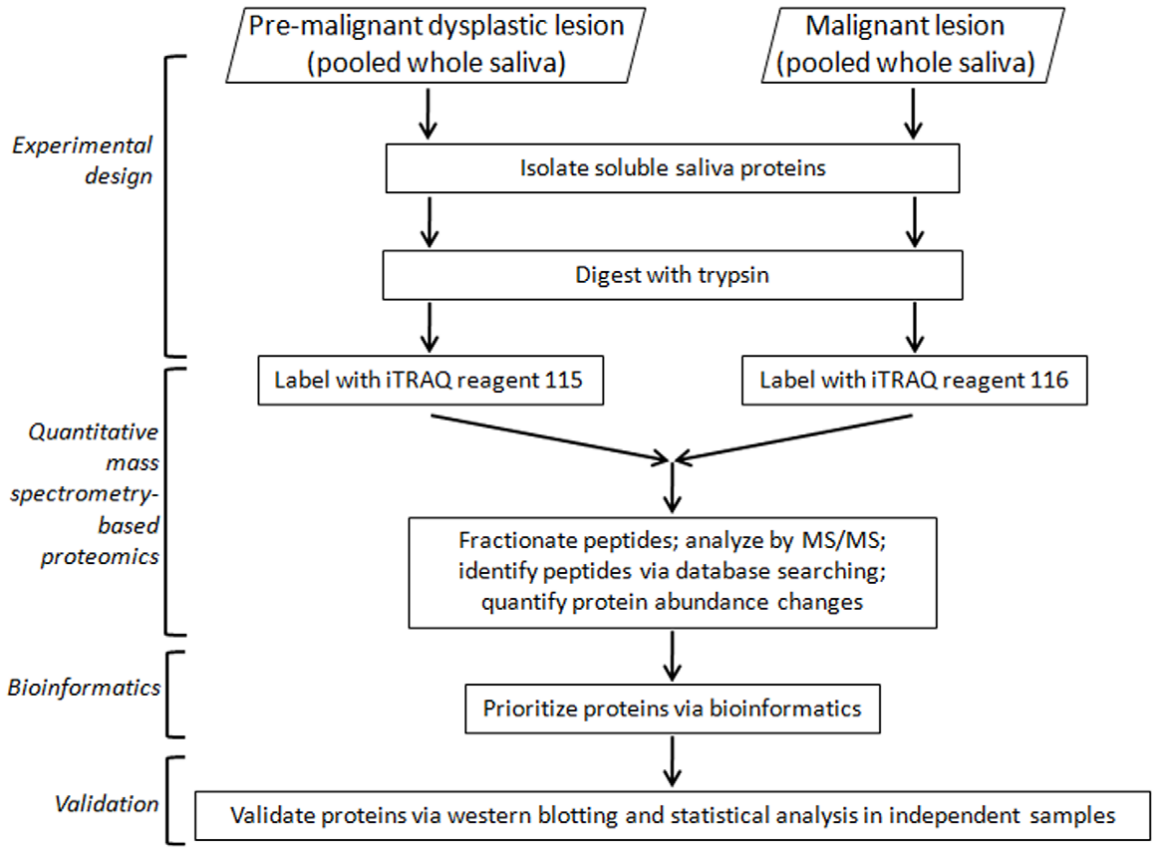
Biomarker discovery strategy. The parts making up our integrated strategy are shown and described in the text.

For biomarker discovery, we employed a strategy implementing several parts (**Figure 1**): experimental design, quantitative mass spectrometry-based proteomics, bioinformatics, and validation. Experimental details on the parts of our strategy are described in Materials and Methods. We included for analysis saliva samples collected from subjects at two different stages of oral cancer development. These stages, detailed at the top of **Figure 1**, follow the known course of development of oral cancer, transitioning from a pre-malignant dysplastic lesion to a malignant lesion in the form of OSCC[17]. **Supplementary Table S1** contains relevant information on all of the subjects whose saliva was analyzed in our studies.

Our proteomics analysis identified a rich catalogue of 855 total proteins complete with their relative abundance levels compared between different stages of oral cancer development. Our catalogue (**Supplementary Table S2**) was rigorously filtered ensuring estimated spectral false positive rates below 1% and minimal redundancy (see Materials and Methods for details). Of the quantified proteins, 198 proteins showed significant abundance differences between pooled subjects with pre-malignant and malignant oral lesions.

### Bioinformatics for prioritizing and selecting candidate biomarkers

Because validating proteins is time-consuming and expensive, we could not possibly validate the almost 200 differentially abundance proteins in our catalog. Instead, we sought to prioritize them, and from those assigned highest priority select candidate biomarkers proteins needing further validation. To that end, we used the Ingenuity Pathway Analysis (IPA) tool to group into functional pathways those proteins showing abundance differences between the subjects with pre-malignant lesions and those with malignant lesions. We hypothesized that such grouping would reveal proteins within our catalogue from functional pathways with possible ties to cancer progression, which we would assign high priority. As a control, 198 randomly selected proteins showing no significant abundance difference between the two subject groups were also analyzed. Through this analysis pathways associated with the transition from pre-malignancy to malignancy would be significantly represented in the dataset of proteins changing in abundance, but not in the dataset of control proteins.

**Figure 2:**
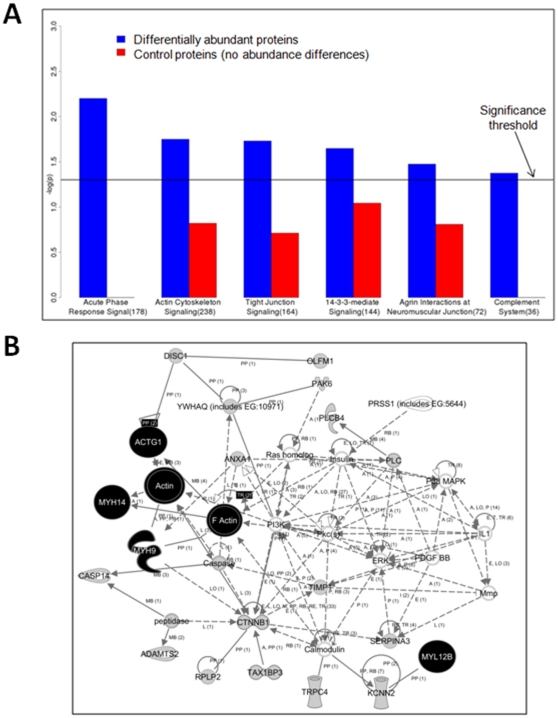
Bioinformatic analysis of differentially abundant proteins. **A.** Significantly represented pathways of differentially abundant proteins compared to control proteins (those showing no abundance differences) grouped by IPA. Pathways with bars rising above the significance threshold are those containing sufficiently high numbers of proteins from our proteomic data such that they are deemed highly represented in a statistically significant manner (See Materials and Methods for details on significance calculations). Numerical values in parentheses for each pathway are the total number of proteins in the Ingenuity Pathway database mapping to that pathway. **B.** A significantly represented protein network (P = 1×10^−35^) determined by IPA on differentially abundant soluble saliva proteins. Myosin subunits and actins are highly represented in this network and shaded in black. Other differentially abundant proteins identified in our proteomics study are shaded in gray; proteins not identified in our proteomics study are not shaded.

Our analysis showed the three most significantly represented pathways to be acute phase response, followed by actin cytoskeleton signaling and tight junction signaling (**Figure 2**). The inclusion of acute phase pathway proteins in our data might be expected, given their role as responders to inflammation, a condition that is central to oral cancer progression[6], [10]. However, their activation within the oral cavity in response to a variety of conditions other than cancer[18], [19] limited their value in prioritizing proteins for validation.

Conversely, the next two most significant categories, actin cytoskeleton signaling and tight junction signaling, were assigned high priority given their role in transforming cells from a pre-malignant state to an invasive, malignant state[20], [21]. Closer inspection of those proteins revealed that myosin subunits were highly represented, with all of these subunits showing increases in abundance between the pre-malignant and malignant pooled saliva samples from our proteomics data. We therefore selected myosin as a candidate for further validation. Given the close functional relationship of myosin with actin, and their joint role in cell invasion in cancer progression[22], we expected that actin might show a similar change in abundance. Inspection of our catalogue revealed that beta actin, a main cytoskeletal actin isoforms did increase by a modest amount (normalized abundance ratio of 1.2), although it did not pass our criteria as being a significant increase. Given this at least modest increase in abundance along with its functional relationship with myosin, we also selected actin for further validation as a candidate biomarker. As a follow-up analysis, we also performed network analysis using IPA on the differentially abundant proteins, and a network containing actin and myosin subunits was among the most significant returned (P = 1×10^−35^, **Figure 2B**), further supporting the prevalence of actin and myosin proteins among those showing abundance differences.

In addition to the myosin and actin, we also prioritized proteins from our catalogue via manual inspection seeking to select other candidate biomarker proteins warranting validation. Standing out were 28 different keratin proteins identified and quantified in our proteomics analysis, with the majority of these showing significant increases in abundance between the pre-malignant and malignant sample pools. Among the keratins, we selected keratin 19 for validation, given past descriptions of its increased expression in OSCC tissues[23], [24]. We also observed that catalase, recently described as a saliva protein whose abundance change distinguishes healthy individuals from those with malignant oral lesions[9], and the protein S100A7, recently described as a biomarker distinguishing healthy and malignant oral tissue biopsies[7], were both modestly increased in abundance between the pre-malignant and malignant sample pools in our proteomic catalogue. We therefore selected both of these proteins for validation.

### Validation and statistical analysis of candidate biomarkers

Candidate biomarkers (myosin, actin, S100A7, keratin 19 and catalase) were validated via two rounds of quantitative western blotting. Initially, we screened each protein in a small number of independent samples from three pre-malignant and three malignant subjects, seeking to confirm our proteomic results and determine those showing the most significant and consistent quantitative differences, which would then be selected for validation in a larger number of patient samples. From this screen, S100A7, catalase and keratin 19 did not show consistent differences between pre-malignant and malignant subjects to merit further validation (data not shown). Actin and myosin on the other hand showed clear and consistent increases in their abundance between samples from individuals with pre-malignant and malignant oral lesions. We therefore selected these two proteins for validation and statistical analysis in a larger number of patient samples.

For actin and myosin validation, we analyzed by western blot saliva samples collected from twelve subjects with pre-malignant oral lesions and twelve subjects with malignant oral lesions (see **Supplementary Table S1** for information on subjects). None of these subjects were used in our initial quantitative proteomics analysis. In order to quantitatively compare western blotting results measuring actin and myosin abundance levels in pre-malignant or malignant subjects run on different gels, we used a common reference sample on all blots, prepared by pooling saliva from eleven healthy donors. For all western blots, the same amount of protein from this reference sample and each subject was loaded on the gel, and the signal intensity from either the actin or myosin band from each subject normalized against the signal intensity derived from the reference. To guard against potential errors in sample loading and ensure the veracity of our findings, blots for actin and myosin were performed in triplicate, and normalized intensities for each protein averaged for each subject across the three blots. **Supplementary Figure S1** shows all the images of all western blotting results for myosin and actin.

**Figure 3:**
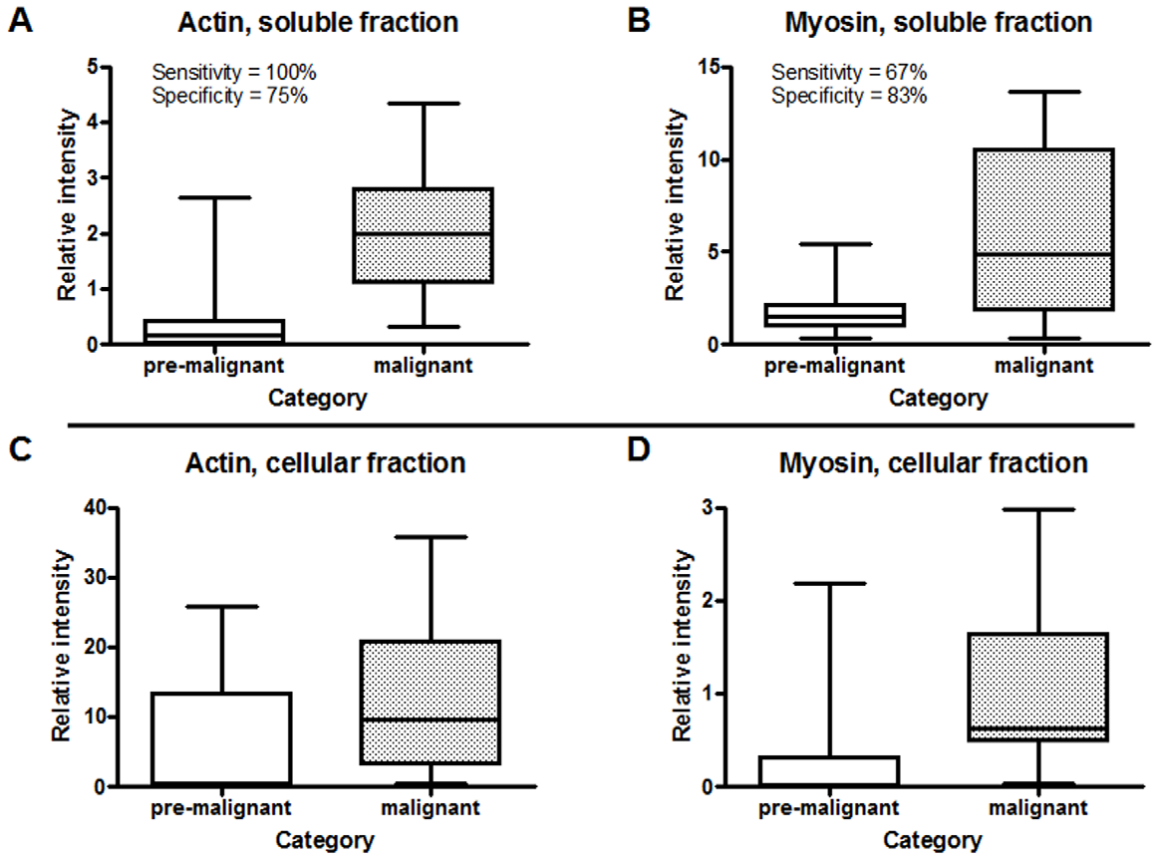
Summary of western blot validation results. Box plots of actin and myosin western blot data in soluble saliva (panels A and B, mean of twelve samples tested in each subject group; the ratio for each sample being the mean of three technical replicates) and exfoliated cells in the same saliva samples (panels C and D, mean of twelve samples tested in each subject group). Each plot displays the median (solid horizontal line within each box), quartiles (area if each box), and range of the data (bars extending from each box). Western blots were performed for actin (A, C) and myosin (B, D).

**Figure 3** (panels A and B) shows a plot of the average normalized intensity ratio from the twelve pre-malignant and malignant subjects for actin and myosin, respectively. Both proteins showed a clear increase in their salivary abundance in subjects with malignant oral lesions compared to those with pre-malignant lesions. Using the relative abundance levels compared to the reference measured for each subject, sensitivity and specificity values for distinguishing between subjects with pre-malignant or malignant oral lesions were 100% and 75%, respectively, for actin (p = 0.002) and 67% and 83%, respectively, for myosin (p<0.00001).

To increase confidence in our results, we sought additional experimental evidence confirming the source of our observations for actin and myosin in soluble saliva being their differential expression in pre-malignant or malignant cells. Given that actin and myosin are intracellular proteins, we assumed that the main source of these proteins in saliva would be epithelial cells within the oral cavity. We hypothesized that if our observed soluble saliva abundance differences in these two proteins were truly a consequence of the transformation of cells within oral lesions from a pre-malignant state to a malignant state, their relative abundance levels in exfoliated epithelial cells collected in saliva from the same subjects would mirror those observed in the soluble saliva. Our past results[11], [25] have suggested that the exfoliated cells present in saliva contain diagnostically valuable cells emanating directly from the oral lesion supporting our reasoning for their analysis to test our hypothesis.

To test our hypothesis, we performed a western blot for actin and myosin on proteins from exfoliated cells contained in the saliva of the same pre-malignant and malignant subjects used for validations described above. Again, we normalized the intensities of actin and myosin in each subject sample against a common reference sample of proteins derived from exfoliated cells in a saliva sample from pooled healthy donors. **Supplementary Figure S1** shows all the images of all western blotting results for the cellular samples. **Figure 3** **(panels C and D)** shows plots of normalized actin and myosin western blot intensities averaged across the pre-malignant and malignant subjects. Both proteins showed overall increases in normalized abundance levels in subjects with malignant oral lesions compared to those with pre-malignant lesions. These findings support the assertion that our observations for actin and myosin in soluble saliva are a direct consequence of differences in their cellular abundances.

## Discussion

In this study we sought to discover and validate protein biomarkers of oral cancer in the soluble fraction of whole saliva. Our study is the first to use advanced quantitative proteomics to compare saliva collected from subjects with pre-malignant oral lesions to saliva collected from subjects with malignant oral lesions. One recent proteomic study[9] compared saliva from subjects with no oral lesions to saliva from subjects with malignant oral lesions. Although interesting, these results have limited relevance to clinicians who are challenged mainly by distinguishing pre-malignant from malignant oral lesions, rather than asymptomatic healthy subjects with no lesions from those with malignancy. The protein catalase provides an example of this limitation. Catalase was significantly increased in abundance between healthy subjects and those with malignant lesions in a recent study by Wong and colleagues[9]. However, we found that catalase did not reliably distinguish between subjects with pre-malignant lesions and those with malignancy. Including subjects with pre-malignant oral lesions allowed us to identify those candidate protein biomarkers with the highest potential value for solving a critical problem in the clinical management of oral cancer.

Owing to the uniqueness of our study, our protein catalogue (**Supplementary Table S2**) may serve as a valuable guide to others seeking to characterize salivary protein biomarkers of oral cancer. Given the time and expense limitations, we were only able to validate a relatively small number of the almost 200 differentially abundant proteins in our catalogue. It is very possible that other promising biomarkers exist among this group of differentially abundant proteins which we were unable to include in validation studies. These may be identified in ongoing studies by others, and when compared to our catalogue, bolster confidence as candidates for further validation.

In our study, actin and myosin were the most promising candidate saliva biomarkers identified. Both proteins displayed increases in their abundance levels in soluble saliva from subjects with malignant lesions, compared to those with pre-malignant lesions. Additionally both proteins showed abundance increases in the exfoliated cells from the saliva of the same subjects. These latter findings provided critical evidence that the differences observed in the soluble fraction of saliva are most likely a direct result of protein differences occurring within the epithelial cells during the transition from a pre-malignancy to malignancy. Importantly, we did not observe an increase in the amount of exfoliated cells in malignant salivas compared to pre-malignant salivas (data not shown), suggesting that the observed increases in protein abundance are due to increased actin and myosin expression within the exfoliated cells with the onset of invasive oral malignancy, and not simply an increase in the amount of cells in saliva.

In addition to our empirical evidence, previous descriptions of the role of actin and myosin in the mechanism underlying the transformation of pre-malignant epithelial cells to a malignant state also support our findings. Actin and myosin are key cytoskeletal proteins enabling cell motility and invasion, behavior central to epithelial tumorigenesis[22], [26]. Indeed, knockdown of regulators of actin and myosin which causes their decreased expression results in loss of cell migration and reduced invasion in nasopharogeal carcinoma[27]. Consistent with our findings, actin isoforms have been observed to be increased in abundance in *in vitro* tumor cell migration studies[28], invasive basal cell carcinoma[29], cervical squamous cell carcinoma[30], esophageal squamous cell carcinoma[31], as well as invasive OSCC[32]. Likewise, increases in myosin abundance have been reported in proteomic studies of OSCC tissue[33], although other studies seem to contradict these findings[34].

Our calculated sensitivity and specificity values respectively of 100% and 75% for actin, and 67% and 83% for myosin, are at least comparable to published values of other non-invasive detection methods currently used for distinguishing pre-malignant and malignant oral lesions. A recent review by Lingen *et al.* critiques the methods currently used by clinicians[3]. For example, our results compare favorably to conventional oral examination by an expert clinician, which, depending on the study cited, has sensitivities as low as 60% and specificities as low as 75%[3], [35]. Compared to this conventional method, a quantitative test based on abundance levels of salivary actin and/or myosin could have the added advantage of eliminating the subjective nature of the clinical exam. Our results also compare well to the popular light-based detection methods (e.g. ViziLite, VELscope) which, again depending on the study, generally perform well, although specificity values as low as 14% have been reported[36]. We did evaluate the possibility of combining the relative abundance levels of actin and myosin as a means for improving our ability to distinguish pre-malignant and malignant lesions. However we found that the combining the data did not significantly improve our sensitivity and specificity.

In conclusion, via a proteomics study we have identified actin and myosin as promising salivary biomarkers of oral cancer. More rigorous determination in a larger number of samples of sensitivity and specificity values for both proteins is necessary to better understand their potential as biomarkers for widespread clinical testing. However, our results illustrate their promise for development into a simple and cost-effective test for oral cancer, with potential implementation using emerging point-of-care nano-scale devices for clinical diagnostics[37].

## Supporting Information

Table S1 Information on subjects used in discovery and validation studies.
(0.09 MB PDF)

Table S2 Quantitative Proteomic Catalogue of Whole Saliva Soluble Fraction.
(1.22 MB XLS)

Figure S1 Western blotting images from validation studies. Lanes marked with “R” denotes the reference sample; each numbered lane is a sample from a different individual with either a pre-malignant or malignant oral lesion. Approximate positions of the molecular weight standards, in kDa, are included.
(2.04 MB TIF)
