# Supplementary material for: Quantitative Proteomics Reveals Myosin and Actin as Promising Saliva Biomarkers for Distinguishing Pre-Malignant and Malignant Oral Lesions
Source: PLoS One. 2010 Jun 17;5(6):e11148. doi: 10.1371/journal.pone.0011148 (PMC2887353; doi:10.1371/journal.pone.0011148)
Supplement: Table S1 — Information on subjects used in discovery and validation studies. (0.09 MB PDF) [file pone.0011148.s001.pdf]

Supplementary Table S1

Relevant information on subjects used in discovery and validation experiments

Subject*	Subject group/experiment	Gender	Age	Lesion location	Dysplasia grade	Ulceration state	Smoker?
1	Pre-malignant/discovery	M	67	tongue	Severe	Severe	no
2	Pre-malignant/discovery	F	74	tongue	Severe	Severe	no
3	Pre-malignant/discovery	M	49	tongue	Moderate	Moderate	yes
4	Pre-malignant/discovery	M	52	tongue	Moderate		yes
5	OSCC/discovery	F	52	tongue	N/A		no
6	OSCC/discovery	M	65	tongue	N/A		yes
7	OSCC/discovery	F	44	tongue	N/A		no
8	OSCC/discovery	M	75	tongue	N/A		no
9	Pre-malignant/validation	F	67	tongue	Severe		no
10	Pre-malignant/validation	F	52	tongue	Moderate		no
11	Pre-malignant/validation	M	55	tongue	Severe		yes
12	Pre-malignant/validation	F	67	tongue	Severe		no
13	Pre-malignant/validation	M	66	tongue	Severe	Severe	no
14	Pre-malignant/validation	F	49	tongue	Early	Moderate	no
15	Pre-malignant/validation	M	67	tongue	Severe	Moderate	no
16	Pre-malignant/validation	F	65	tongue	Early		no
17	Pre-malignant/validation	F	75	tongue	Severe	Severe	no
18	Pre-malignant/validation	M	44	buccal	Severe	Moderate	yes
19	Pre-malignant/validation	M	70	tongue	Severe	Moderate	yes
20	Pre-malignant/validation	M	65	tongue	Moderate		yes
21	OSCC/validation	M	64	tongue	N/A		yes
22	OSCC/validation	F	49	tongue	N/A		no
23	OSCC/validation	M	66	gum	N/A	Moderate	yes
24	OSCC/validation	F	77	tongue	N/A	Moderate	yes (former)
25	OSCC/validation	M	60	tongue	N/A		yes
26	OSCC/validation	M	55	tongue	N/A	Moderate	yes
27	OSCC/validation	M	57	tongue	N/A	Severe	no
28	OSCC/validation	F	47	tongue	N/A		yes
29	OSCC/validation	M	27	tongue	N/A		no
30	OSCC/validation	F	55	tongue	N/A	Severe	no
31	OSCC/validation	M	86	tongue	N/A		yes
32	OSCC/validation	F	38	tongue	N/A		no

*Subjects 1-4, and 5-8 were pooled together for proteomics discovery experiments. Subjects 9-32 were analyzed individually in validation experiments. N/A = not applicable
