# Supplementary figures and images for: Quantitative Proteomics Reveals Myosin and Actin as Promising Saliva Biomarkers for Distinguishing Pre-Malignant and Malignant Oral Lesions
Source: PLoS One. 2010 Jun 17;5(6):e11148. doi: 10.1371/journal.pone.0011148 (PMC2887353; doi:10.1371/journal.pone.0011148)

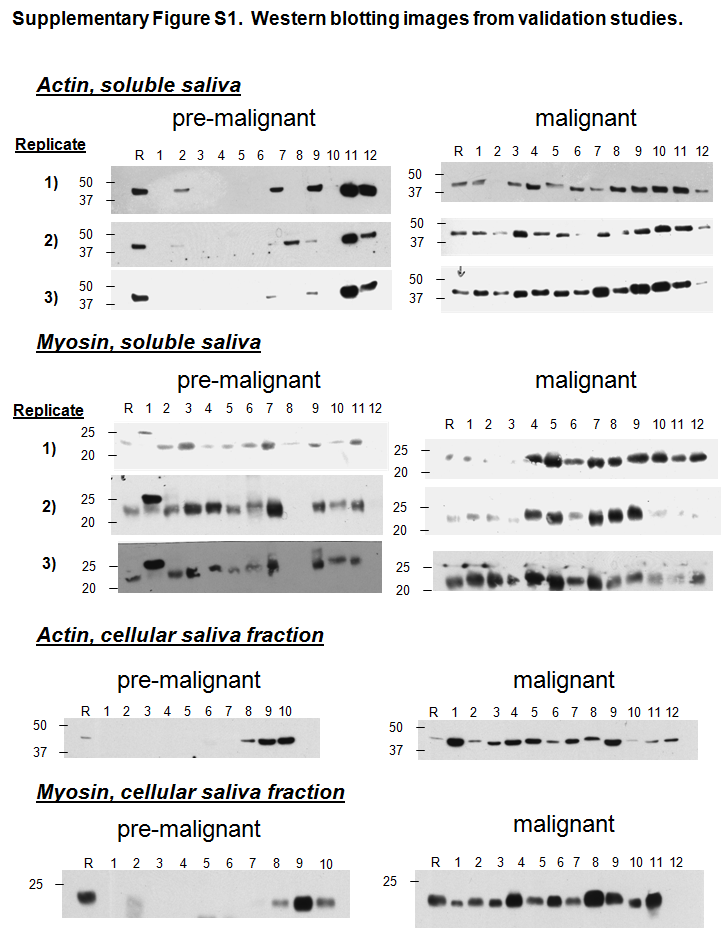

Supplement: Figure S1 — Western blotting images from validation studies. Lanes marked with “R” denotes the reference sample; each numbered lane is a sample from a different individual with either a pre-malignant or malignant oral lesion. Approximate positions of the molecular weight standards, in kDa, are included. (2.04 MB TIF) [file pone.0011148.s003.tif]
